# Dietary factors associated with metabolic syndrome in Brazilian adults

**DOI:** 10.1186/1475-2891-11-13

**Published:** 2012-03-14

**Authors:** Erick Prado de Oliveira, Kátia Cristina Portero McLellan, Liciana Vaz de Arruda Silveira, Roberto Carlos Burini

**Affiliations:** 1 Center for exercise metabolism and nutrition (CeMENutri), Department of Public Health, Botucatu School of Medicine (UNESP), Botucatu, Brazil; 2 Department of Pathology, Botucatu School of Medicine (UNESP), Botucatu, Brazil; 3 Department of Bioestatistic, Bioscience Institute (UNESP), Botucatu, Brazil; 4 CeMENutri - Faculdade de Medicina, Depto. de Saúde Pública (FMB - UNESP), Distrito de Rubião Jr, s/n° 18.618-970, Botucatu, SP, Brazil

**Keywords:** Diet, Metabolic Syndrome, Diet variety, Fruit intake, Saturated fat

## Abstract

**Background:**

Metabolic Syndrome (MS) is defined as the association of numerous factors that increase cardiovascular risk and diet is one of the main factors related to increase the MS in the population. This study aimed to evaluate the association of diet on the presence of MS in an adult population sample.

**Methodology:**

305 adults were clinically screened to participate in a lifestyle modification program. Anthropometric assessments included waist circumference (WC), body fat and calculated BMI (kg/m^2^) and muscle-mass index (MMI kg/m^2^). Dietary intake was estimated by 24 h dietary recall. Fasting blood was used for biochemical analysis. MS was diagnosed using NCEP-ATPIII (2001) criteria with adaptation for glucose (≥ 100 mg/dL). Logistic regression (Odds ratio) was performed in order to determine the odds ratio for developing MS according to dietary intake.

**Results:**

An adequate intake of fruits, OR = 0.52 (CI:0.28-0.98), and an intake of more than 8 different items in the diet (variety), OR = 0.31 (CI:0.12-0.79) showed to be a protective factor against a diagnosis of MS. Saturated fat intake greater than 10% of total caloric value represented a risk for MS diagnosis, OR = 2.0 (1.04-3.84).

**Conclusion:**

Regarding the dietary aspect, a risk factor for MS was higher intake of saturated fat, and protective factors were high diet variety and adequate fruit intake.

## Background

Metabolic Syndrome (MS) is defined as the association of numerous factors that increase cardiovascular risk [1], such as dyslipidemia, abdominal obesity, systemic arterial hypertension and impaired fasting glycemia. All these altered factors are related to a prothrombotic and proinflammatory condition [2,3].

MS prevalence in the United States is 21.8% [4], and it has increased strongly over the last few years [5]. In Brazil, no data have been reported for the national prevalence, but some studies have shown that MS prevalence at a regional level is approximately 15.8 to 48.3% [6,7].

It is known that MS etiology includes genetic, metabolic and environmental factors [8]. Among environmental factors, diet is one of the main aspects related to the increase in MS in the population [9].

The western diet, which is characterized by the intake of processed meat, refined grains, fried meat and sugar-based desserts, is directly associated with the risk of MS [10]. On the other hand, the Mediterranean diet has been inversely associated with MS development [11]. Epidemiological studies and control cases have shown a protective effect from dietary patterns characterized by the intake of vegetables, fruit, legumes, whole grain, fiber, fish, lean meat, poultry and fat-free dairy products [12,13].

Studies have shown that some dietary components directly influence MS, both as protective factors [11,14] and as risk factors [15,16]. However, no Brazilian study has investigated the influence of dietary intake on MS, only on isolated MS components [17-19]. Therefore, the aim of this study was to evaluate dietary influence on MS in an adult population sample.

## Methods

### Individuals

This is a descriptive cross-sectional study conducted from 2004 to 2008 on a sub-group of participants clinically selected and enrolled for the lifestyle modification program "*Mexa-se Pró-Saúde*" (Move for Health), described in elsewhere [1]. All individuals that had all the MS components data were included to the study. A convenience sample were consisted of three hundred and five individuals, with MS (39.7%) or not, males and females, aged > 35 years, were evaluated. All the participants have never participated of any program of lifestyle change before and signed an informed-consent form, which, conjointly with the project, was approved by the Research Ethics Committee (document no. CEP 3271-2009) of the Botucatu School of Medicine (FMB - UNESP).

### Marital status

Marital status was classified as single + divorced vs married + cohabiting. Educational level was evaluated as complete and incomplete secondary and college education vs illiterate, complete and incomplete primary education. Family income ≥ 6 minimum salaries vs < 6 minimum salaries.

### Body composition

The anthropometric assessment consisted of measuring body weight and height, according to the previously described procedures [20], followed by BMI estimation. Waist circumference (WC) was measured by a non-extensible and non-elastic millimeter-graded measuring tape. Measurement was made on the mid-point between the last intercostal space and the iliac crest. The reference values proposed by NCEP-ATP III [21,22] were used and WC larger than 88 cm for females and 102 for males was considered to be increased [21].

Bioelectric impedance (Biodynamics^®^, model 450, USA) was used to determine the percentage of body fat (%BF) and muscle mass (kg). Segal *et al *(1988) equation was used to calculated the %BF [23]. The muscle mass (kg) was obtained using the Janssen *et al*., (2000) equation [24] and the muscle mass index (MMI) was calculated as MM (kg)/height^2^.

### Clinical evaluation of arterial blood pressure

Systolic and diastolic arterial blood pressure was evaluated with the individual in the seated position according to the procedures described by the V Brazilian Guidelines on Arterial Hypertension [25]. Values of systolic blood pressure ≥ 130 mm Hg and/or diastolic blood pressure ≥ 85 mm Hg were considered abnormal.

### Biochemical analyses

For biochemical analyses, the individuals were submitted to blood sample collections after nocturnal fasting (8 a 12 hours) by standard venipuncture. Glucose, triglycerides (TG) and high-density lipoprotein cholesterol (HDL-c) concentrations were quantified in serum by the dry chemistry method. The classification of normality levels followed NCEP-ATPIII [21,22].

### MS diagnosis

The individuals were diagnosed as having MS according to NCEP-ATP III [21,22], with glycemia levels adapted for 100 mg/dL [26].

The five components used were plasma concentrations of triglycerides, HDL-c and fasting glucose, arterial hypertension and abdominal circumference. MS was diagnosed when three or more of these components were altered.

### Dietary intake assesment

The 24 h dietary recall was used to assess food intake [27]. Dietary data obtained in homemade measurements were converted into grams and milliliters to permit chemical analysis of food intake. The centesimal composition of foods present in the records was calculated using NutWin^® ^(2002) software, version 1.5. Foods not found in the software were added from diverse composition tables and food labels [28,29]. Diet quality was evaluated using the Adapted Healthy Eating Index (HEI) [30] and evaluated groups were based on portions recommended by the Adapted Food Pyramid [31].

### Statistical analysis

The analyses were performed by using SAS, version 9.1, and STATISTICA 6.0. The data were described as mean ± SD and median and interquartile interval. Sample normality was tested by means of the Shapiro-Wilk test. For comparison of individuals with or without MS, the t test was used for normal distribution. Wilcoxon analysis for not-normal distribution, and the Chi-square test for categorical variables. In order to correlate the dietary variables with the number of MS components and with each component isolated, partial pearson's correlation was used (adjusted for gender, age, total caloric value (TCV) and BMI). Logistic regression (odds ratio) was performed, with a confidence interval (CI) of 95%, in order to evaluate the odds ratio for developing MS according to dietary intake. Two models were used, and the first was adjusted for gender and age; the second was additionally adjusted for TCV and BMI. The results were discussed based on the level of significance of *p *< 0.05.

## Results

The individuals with MS were older and showed poorer education than those without MS. Income, marital status and gender were similar in both groups. As regards body composition, the individuals with MS showed higher adiposity (> BMI and %BF) and higher MMI. Additionally, lower intake of fruit servings and smaller variety was observed in the diet of individuals with MS (Table 1).

**Table 1:** Demographic, body-composition and dietary characteristics of individuals with and without the Metabolic Syndrome (MS).

	Without MS (n = 184)	With MS (n = 121)	*p*
**Age (years)**	54.2 ± 10	55.8 ± 10	< 0.0001

**% females**	73.4	73.5	0.96

**Marital status (single + divorced)**	47.4%	57.5%	0.08

**Education (illiterate; complete and incomplete elementary school)**	46.7%	61.1%	0.01

**Income (> 6 minimum salaries)**	44%	37.5%	0.29

**BMI (kg/m^2^)**	27.5 ± 4.7	31.5 ± 4.7	< 0.0001

**%Body Fat**	30.6 ± 8	36.5 ± 5	< 0.0001

**Muscle Mass Index**	8.2 ± 1.5	8.8 ± 1.7	0.0004

**TCV (kcal)**	1520 ± 578	1521 ± 708	0.99

**% Carbohydrate**	52.3 ± 9.2	50.5 ± 9.2	0.08

**% Protein**	18.4 ± 5.4	18.9 ± 6.2	0.44

**% Total lipid**	29.2 ± 8.6	30.7 ± 8.1	0.14

**% Saturated lipid**	7.5 (5.2-10.3)	7.6 (5.7-9.6)	0.66

**% Monounsaturated lipid**	8.3 (6.3-10.6)	8.9 (6.8-11.7)	0.18

**% Polyunsaturated lipid**	6.8 (4.8-9.2)	7.5 (5.4-9.7)	0.09

**Cholesterol (mg)**	133 (89-214)	138 (87-204)	0.69

**Fibers (g)**	13.6 (8.6-19)	12.6 (7.5-19)	0.31

**HEI (score)**	82.7 ± 14	81.2 ± 13	0.32

**Oil (number of servings)**	1.5 (1.0-2.5)	1.5 (1.0-3.0)	0.63

**Grains (number of servings)**	3.0 (2.4-4.0)	3.0 (2.0-4.0)	0.62

**Fruit (number of servings)**	2.5 (0.4-4.5)	1.4 (0.0-3.5)	0.01

**Vegetables (number of servings)**	1.5 (0.5-3.0)	1.5 (0.5-2.6)	0.8

**Legumes (number of servings)**	1.0 (0.3-2.0)	1.0 (0.4-1.5)	0.32

**Dairy products (number of servings)**	1.4 (0.5-2.0)	1.0 (0.5-2.0)	0.64

**Meat (number of servings)**	1.5 (1.0-2.5)	1.5 (1.0-2.0)	0.72

**Sugar (number of servings)**	1.0 (0.06-2.0)	1.0 (0.4-2.0)	0.6

**Variety**	12.0 (10-15)	11.0 (9.0-15)	0.026

BMI = Body Mass Index; TCV = Total Caloric Value; HEI = Healthy Diet Index; LDL-c = Low-Density Lipoprotein cholesterol

Data expressed in mean ± SD or median and interquartile interval

A positive and weak (r < 0.3) correlation was observed among the number of altered MS components, sugar and total lipid intake whereas a negative and week correlation was found between carbohydrate % and the number of fruit servings (*p *< 0.05). All the other dietary variables did not correlate (*p *> 0.05).

It was observed that adequate intake of fruit servings and diet variety ≥ 8 food items showed a protective effect for SM. Intake higher than 10% of TCV of saturated fat was found to be a risk factor (Table 2).

**Table 2:** Odds ratio for MS according to dietary intake.

	MS	
	
	Odds ratio (95% CI)	Odds ratio (95% CI)
	**Model 1**	**Model 2**

**%Carbohydrate (< 50 vs > 60%)**	1.6 (0.72-3.57)	0.64 (0.28-1.45)

**%Carbohydrate (50-60 vs > 60%)**	1.2 (0.52-2.8)	0.65 (0.27-1.59)

**%Protein (≤ 15 vs > 15)**	0.6 (0.31-1.15)	1.59 (0.80-3.14)

**%Total lipid (≤ 35 vs > 35)**	0.92 (0.49-1.7)	0.91 (0.48-1.72)

**%Saturated lipid (> 10% vs ≤ 10%)**	0.62 (0.34-1.17)	2.0 (1.04-3.84)*

**%Polyunsaturated lipid (≤ 10% vs > 10%)**	1.22 (0.66-2.27)	1.25 (0.66-2.39)

**Cholesterol (≤ 300 mg vs > 300 mg)**	0.91 (0.38-2.19)	0.69 (0.25-1.91)

**Fibers (≥ 20 g vs < 20 g)**	0.75 (0.38-1.46)	0.67 (0.32-1.38)

**HEI (good vs. bad)**	0.91 (0.27-3.07)	0.88 (0.24-3.19)

**Oil servings (≤ 2 vs > 2)**	1.3 (0.73-2.34)	1.18 (0.60-2.32)

**Grains servings (≥ 5 vs < 5)**	1.15 (0.54-2.47)	1.40 (0.58-1.41)

**Fruit servings (≥ 3 vs < 3)**	0.53 (0.29-0.97)*	0.52 (0.28-0.98)*

**Vegetable servings (≥ 4 vs < 4)**	1.0 (0.24-4.17)	0.97 (0.22-4.27)

**Legume servings (≥ 1 vs < 1)**	0.67 (0.39-1.17)	0.69 (0.39-1.24)

**Dairy product servings (≥ 3 vs < 3)**	1.45 (0.60-3.44)	1.6 (0.64-4.03)

**Meat servings (≤ 2 vs > 2)**	1.24 (0.65-2.38)	1.22 (0.58-2.55)

**Sugar servings (≤ 2 vs > 2)**	1.01 (0.52-1.96)	0.82 (0.38-1.78)

**Dietary variety (≥ 8 vs < 8)**	0.41 (0.18-0.91)*	0.31 (0.12-0.79)*

**Model 1**: Adjusted for gender and age

**Model 2**: Model1+ BMI and TCV

*p < 0.05

In order to explain the protective factor of diet variety for MS, a correlation was made between diet variety and the intake of macronutrients and groups on the food pyramid to evaluate what is the intake profile of individuals who ingested a higher number of different food types. Hence, a positive correlation between diet variety and the intake of vegetables (r = 0.34; *p *< 0.05), fruit (r = 0.32; *p *< 0.05), fibers (r = 0.22; *p *< 0.05), dairy products (r = 0.20; *p *< 0.05) and HEI (r = 0.20; *p *< 0.05) was observed, and a negative correlation for the intake of legumes (r = -0.11; *p *< 0.05), meat (r = -0.15; *p *< 0.05) and grains (r = -0.19; *p *< 0.05) was found. There wasn't a significant correlation for the intake of macronutrients, oil servings and sugar.

## Discussion

The main finding of the present investigation was the protective effect of fruit intake and increase in diet variety and the harmful effect of saturated fat intake on the chances of developing MS.

Levy-Costa *et al*. (2005) observed the presence of excessive sugar and saturated fat intake and insufficient fruit and vegetable intake in the Brazilian diet [32]. Our results appear to confirm that this dietary habit is directly associated with the higher risk for MS in this population.

Other studies have shown a protective effect from fruit intake against MS [33-35], indicating that such intake is related to the reduction of almost all MS components (TG, hypertension, WC and glucose) [18,34]. The main protective factor from fruit probably results from the intake of soluble fibers [33]. The intake of this fiber type can reduce glycemia from lower absorption and digestion of carbohydrates, thus reducing insulin secretion, in addition to reducing dyslipidemia [36]. Additionally, the potassium present in this food type can also reduce arterial pressure [37]. The intake of food with low glycemic loads, such as fruit, is related to smaller WC [38], possibly due to the greater satiation [39] and lower energy density provided by this food type [40].

When the diet is evaluated as a whole, the food patterns that are characterized by large amounts of fruit, vegetables and whole grains, such as the Mediterranean diet, are inversely associated with MS prevalence [11,41-43] whereas the western diet, characterized by low fruit intake, is positively associated with MS [10].

Another protective factor identified in the present study was diet variety, evaluated by the number of different food types ingested in 24 h. To our knowledge, this is the first study reporting diet variety as a protective factor against MS development, and further studies are necessary to explain the possible mechanisms involved. Analysis of the results showed that greater diet variety was correlated with a better quality diet (HEI) and with food groups considered to be healthy, such as the intake of vegetables, fruit, fibers and dairy products. These food groups have been reported by other studies as prevalence reducers and protective factors against MS development [33,34,37,39,44,45]. Furthermore, the diet variety could be influenced for the fruit intake isolated, which is also a protector factor for MS. The present study also identified an inverse correlation of diet variety with meat intake, thus showing that this food type increases the chances for MS development [15].

A study conducted with older individuals showed that the inadequate intakes of several nutrients were associated with lower diet variety. It means that recommendations to increase diet variety may be necessary for elderly people to achieve adequate nutrient intakes [46].

Another possible cause for the protective effect of diet variety could be the fact that the individuals whose had the higher diet varied showed greater fractioning during the evaluated day. Studies show that increased fractioning is responsible for decreasing fasting glycemia [47], plasma concentration of postprandial insulin, plasma concentrations of LDL-c and total cholesterol [48]. Additionally, greater fractioning increases postprandial thermogenesis [48] and can foster the negative energy balance, thus providing weight loss in the long term and as a positive factor for MS reduction.

The present study showed increased chances of MS development in individuals who ingest > 10% of saturated fat TCV. This association may have occurred due to the fact saturated fat increases the visceral adipose tissue in greater proportion than other fat types, since it reduces the activation of PGC-1α (PPARγ-coactivator), an important oxidative metabolism regulator. Thus, fatty acid and glucose oxidation is decreased, and accumulation in tissues and circulation is increased [49]. Hence, saturated fat intake would increase the visceral adipose tissue, which contributes to MS development.

Additionally, saturated fat increases insulin resistance (IR) compared to monounsaturated fat [50], because it activates serine kinases, thus inhibiting the insulin phosphorylation cascade, decreasing glucose uptake and increasing glycemia [51]. Another contributing factor to IR is the increase in inflammation, apoptosis and oxidative stress of adipocytes [49].

Some epidemiological studies showed that total dietary fat intake is positively associated with MS in Japanese Brazilian populations [52] and in other countries [16], and this positive association is directly related to saturated fat [53], which is in agreement with our results.

The present study showed that individuals with MS had poorer education, were older and presented higher adiposity (BMI, %BF and WC) and muscle mass (MMI). Another studied reported a positive correlation between BMI and muscle mass, showing that obese individuals present not only higher fat mass, but also show higher muscle mass (kg) [54].

Epidemiological studies have shown the influence of age on MS [55,56], and this occurs due to the fact that ageing is related to body fat mass and lean mass redistribution with rapid increase in visceral intra-abdominal fat, which is proinflammatory and related to insulin resistance [57].

The relation between poorer education and hyperadiposity has been observed by other studies [58]. This relation can be explained by the fact that individuals with poorer education consume less fruit, vegetables and legumes; i.e. their diet is of lower quality [59]. The intake of these food groups were protective factors against MS in the present study.

The present study has some limitations. For this research has used the convenience sample, it do not allow generalizations for the Brazilian population. Furthermore, results from several studies using different food inquiry techniques showed underestimation in intake reports, both in males and females. However, the underestimation is primarily identified among obese individuals and particularly among females [60]. Considering that all the methods that evaluate dietary intake are, to some extent, imperfect and that a gold standard does not exist in nutrition, patient diet was evaluated by analysis of 24-hour recalls. This fact represents the principal limitation in our study, since one 24-hour recalls do not detect dietary intake variations.

## Conclusion

In this study, recommended intake of fruit and high diet variety are protective factors and high intake of saturated fat is a risk factor for metabolic syndrome.

Future proposals for intervention should consider lifestyle changes with adequate eating habits in these individuals.

## Abbreviations

BMI: Body mass index; MS: Metabolic syndrome; WC: Waist circumference; NCEP-ATPIII: National cholesterol education program-adult treatment painel III; HEI: Healthy eating index; TCV: Total caloric value; HDL: High density lipoprotein cholesterol; TG: Triglycerides.

## Competing interests

The authors declare that they have no competing interests.

## Authors' contributions

EPO wrote the manuscript, KCPM corrected and revised the final manuscript. LVAS made the statistical analysis and corrected the manuscript. RCB was the mentor of the work and advisor of the authors. All authors read and approved the final manuscript.
